# Gene masking - a technique to improve accuracy for cancer classification with high dimensionality in microarray data

**DOI:** 10.1186/s12920-016-0233-2

**Published:** 2016-12-05

**Authors:** Harsh Saini, Sunil Pranit Lal, Vimal Vikash Naidu, Vincel Wince Pickering, Gurmeet Singh, Tatsuhiko Tsunoda, Alok Sharma

**Affiliations:** 1 The University of the South Pacific, Laucala Bay, Suva, Fiji; 2 School of Engineering and Advanced Technology, Massey University, Palmerston North, New Zealand; 3 RIKEN Center for Integrative Medical Sciences, Yokohama, 230-0045 Japan; 4 CREST, JST, Yokohama, 230-0045 Japan; 5 Medical Research Institute, Tokyo Medical and Dental University, Tokyo, 113-8510 Japan; 6 Griffith University, Brisbane, Australia

## Abstract

**Background:**

High dimensional feature space generally degrades classification in several applications. In this paper, we propose a strategy called gene masking, in which non-contributing dimensions are heuristically removed from the data to improve classification accuracy.

**Methods:**

Gene masking is implemented via a binary encoded genetic algorithm that can be integrated seamlessly with classifiers during the training phase of classification to perform feature selection. It can also be used to discriminate between features that contribute most to the classification, thereby, allowing researchers to isolate features that may have special significance.

**Results:**

This technique was applied on publicly available datasets whereby it substantially reduced the number of features used for classification while maintaining high accuracies.

**Conclusion:**

The proposed technique can be extremely useful in feature selection as it heuristically removes non-contributing features to improve the performance of classifiers.

## Background

Traditionally, clinical methods are employed to detect cancers such as ultrasonography, X-Ray, Computed Tomography (CT) and Magnetic Resonance Imaging (MRI) [1]. However, many cancers cannot be distinguished easily using traditional approaches. An alternative approach to improve detection is to use analyze microarray gene profiles. In microarray gene profiles, mRNA samples are used to measure the expression level of genes, which can be in the magnitude of thousands. This in turn makes detection and classification of difficult due to the high dimensionality in data [2], therefore, there is a need for computation methods to help improve the classification of cancers using microarray gene profiles.

Generally, computational methods are used to remove non-contributing and noisy dimensions from data while simultaneously trying to maintain a high classification rate [3]. Additionally, class imbalance is an important consideration in classification of biomedical data, and there are techniques [4] which incorporate class distribution within the classification algorithm. Our approach is different in that we separate the classification from data preprocessing where we assume class imbalance is to be handled.

Feature selection and extraction is a well researched topic in biomedical fields, especially in the areas concerning microarray data [5–7]. Several methods have been discussed relating to feature selection for microarray data [6, 8–17] and they can be broadly categorized into two groups, filter based methods and wrapper based methods. In filter based methods, genes are selected prior to training the classification model whereas wrapper based methods involve gene selection within the classification process [5, 18, 19].

The importance of selecting features from gene subsets or groups has recently become popular topic in microarray research [7, 20]. For instance, top-r feature selection proposed by Sharma et. al [20] does provide very good results based on a small subset of genes, however, it should be noted that it has a few drawbacks. Firstly, it is quite computationally expensive, requiring a total number of search combinations between ^*h*+1^ *C*_2_×(*d*/*h*) and (2^*h*^−1)×(*d*/*h*), where *h* is the block size and *d* is the total number of dimensions [20]. Additionally, initial parameter selection is crucial and it greatly affects the final results. Top-r is sensitive to the selection of block size and number of resulting blocks. Selecting ideal value of *h* could be a tricky task and final results are dependent on this value [20]. Lastly, it should be noted that top-r does not fully consider the interaction among features but only amongst the top-r features from each block [5].

In this paper, we consider the classification of the small round blue cell tumor (SRBCT) [21] dataset which has been categorized into 4 types of cancers and has 2308 gene expressions. Khan et al. [1], Tibshirani et al. [21] and Kumar et al. [22] have previously worked on this dataset whereby they have all reported 100% classification accuracies with 96, 43 and 13 genes respectively. While Khan et al. [1] and Tibshirani et al. [21] use the fully-fledged dataset with 2308 genes to perform analysis, Kumar et al. [22] begin their analysis from a reduced set of 96 genes (from Khan et al. [1] findings) to obtain results. Kumar et al. [22] do not use all 2308 genes due to the computational complexity of their approach. Our motivation in this paper is to build upon the approach proposed by Kumar et al. [22] and propose a new method that does not suffer from similar limitations. In the proposed method, we propose a wrapper based method where we commence with the entire feature set from the microarray data without any prior need of feature selection and achieve high classification accuracy with as few features as possible.

Furthermore we validate our approach using the mixed-lineage leukemia (MLL) [23] and lung cancer (LC) [24] datasets. MLL dataset comprises of 3 classes, with each sample containing 12,582 gene expressions. Lastly, LC dataset contains 2 cancer types and each sample comprises of 12,533 gene expressions. We applied gene masking with nearest shrunken centroid classifier to significantly reduce the number of dimensions for the datasets while maintaining 100% accuracies during classification.

## Methods

Gene masking has been derived from genetic algorithm, whereby genetic algorithm is used to search for an optimal gene mask that provides the greatest performance gains while removing the most number of features for the selected classification algorithm. For this study, *Nearest Centroid* and *Nearest Shrunken Centroid* classifiers were used for classification.

### Genetic algorithm

The genetic algorithm (GA) is a heuristic search based algorithm inspired by Darwin’s theory of natural selection. It was first introduced by Holland and it simulates natural processes of evolution, namely selection, crossover and mutation. GA is a competitive search algorithm where evolution of individuals is directed mainly by the principle of “survival of the fittest”. Fitness of an individual is determined by a fitness function and individuals with a higher fitness have a greater bias for contributing to the next generation than their less fit counterparts [25]. More details on GA processes and functions are described in latter sections.

### Nearest centroid classifier

Nearest Centroid Classifier (NCC) is a basic prototype classifier that creates centroids (which is the mean for a particular class) to create a classification model. Samples closest to a centroid is assigned a label of that particular class [21].

In NCC, we compute the class centroid by finding the mean of every feature per class:

1 $$ \bar{x}_{ik} = \sum_{j \epsilon C_{k}}\frac{x_{ij}}{n_{k}}   $$

where *x*_*ij*_ is the value at the *i* ^*th*^ feature of the *j* ^*th*^ sample, *k* denotes the class under consideration and *n*_*k*_ is the number of samples in class *k*. Once the class centroids can calculated, we can predict the class $\hat {k}$ for an unknown sample $\hat {x}$ using:

2 $$ \hat{k} = arg\;{min}_{k \epsilon K}||\bar{x}_{k} - \hat{x}||   $$

### Nearest shrunken centroid classifier

Nearest Shrunken Centroid Classifier (NSCC) [21], is a simple modification of NCC that uses “de-noised” versions of the centroids. Features that are noisy and have little variation from the overall mean are removed during shrinkage. The amount of shrinkage is determined by a constant *Δ*, where a larger value of *Δ* removes a larger number of features. Therefore, it can be stated that this classifier has an “in-built” feature selection mechanism.

In order to perform the shrinkage, firstly, we compute the distance of every feature, *d*_*ik*_, from the overall centroid after standardizing by standard deviation of features within a class. In Eq. 3, *x*_*ij*_ is the value at the *i* ^*th*^ feature of the *j* ^*th*^ sample, *K* is is the total number of classes and *k* denotes the class under consideration. The centroid values for feature *i* in class *k* is $\bar {x}_{ik} = \sum _{j \epsilon C_{k}}\frac {x_{ij}}{n_{k}}$, where *C*_*k*_ denotes the indices of *n*_*k*_ samples in class *k*. Likewise, the overall centroid value at the *i* ^*th*^ feature is $\bar {x}_{i} = \sum ^{n}_{j=1}\frac {x_{ij}}{n}$. Also, *m*_*k*_ is defined as ${m^{2}_{k}} = \frac {1}{n_{k}} - \frac {1}{n}$ and ${s^{2}_{i}} = \frac {1}{n-K} \sum _{k} \sum _{j \epsilon C_{k}} (x_{ij} - \bar {x}_{ik})^{2}$, which is the pooled within-class variance for feature *i*. *s*_0_ was chosen to be the median value of *s*_*i*_.

3 $$ d_{ik} = \frac{\bar{x}_{ik} - \bar{x}_{i}}{m_{k} \times (s_{i} + s_{0})}   $$

Once the distances are computed, we perform the actual shrinkage where every *d*_*ik*_ is reduced by an amount *Δ* in absolute value and is set to zero if its absolute value is less than zero. In Eq. 4, + means we only consider the positive part (*t*_+_=*t* if *t*≥0 otherwise zero).

4 $$ d^{\prime}_{ik} = sign(d_{ik})(|d_{ik}| - \Delta)_{+}   $$

In the above equation, $d^{\prime }_{ik}$ defines the shrunken distances. By using *Δ* as a soft threshold, we are effectively removing features that have little or no variation from the overall centroids. In order to obtain the shrunken class centroids, $\bar {x}^{\prime }_{ik}$, we can rewrite Eq. 3 and substitute *d*_*ik*_ with their shrunken representations $d^{\prime }_{ik}$ (Eq. 5) after which we can predict unknown samples as per Eq. 2.

5 $$ \bar{x}^{\prime}_{ik} = \bar{x}_{i} + m_{k}(s_{i} + s_{0})d^{\prime}_{ik}   $$

## Gene masking

Gene masking is a technique that incorporates evolutionary techniques to reduce the dimensionality of data within the training phase of the classification model. The basic premise of this technique is to heuristically remove non-contributing features in data while training the classifier. The amount of contribution by a feature is determined by its impact on classification accuracy, whereby non-contribution is attributed to features whose removal and/or existence has minimal effect on classification accuracy. By reducing the dimensionality of data, gene masking helps improve classifier performance and reduces the computational complexity of the problem. Moreover, it can be used as a feature isolation technique that allows for the identification of features which contribute the most towards classification.

### Overview

**Fig. 1:**
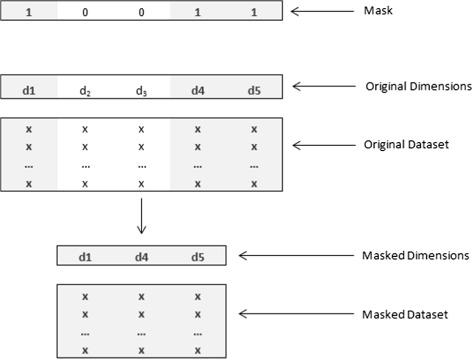
Illustration of gene masking on the original dataset to produce a masked dataset

Gene masking, essentially, is a binary encoded genetic algorithm that generates a template used to represent a chromosome, referred to as a mask, while the individual bits at different indices in the chromosome are annotated as genes. This mask can be visualized as a string of binary digits with length equal to the number of features in data. Each binary digit at a particular index (or a gene in terms of the mask) signifies the presence or absence of the corresponding feature in data. For instance, a problem with five features can represented by a feature vector *[f1 f2 f3 f4 f5]* and a possible gene mask can be *[1 0 0 1 1]*. This mask indicates that features *f2* and *f3* are to be removed from the data and the classification model has to be created using a feature vector comprising of *[f1 f4 f5]*, thus, effectively reducing the dimensionality of data. This process has been depicted in Fig. 1.

In gene masking, the GA processes are unmodified and it goes through its basic set of genetic operations. For each generation, fitness is calculated for every mask in the population. These masks are then exposed to the three GA operators; selection, crossover and mutation. Finally, the best performing mask is chosen after the generation limit is reached in GA.

**Fig. 2:**
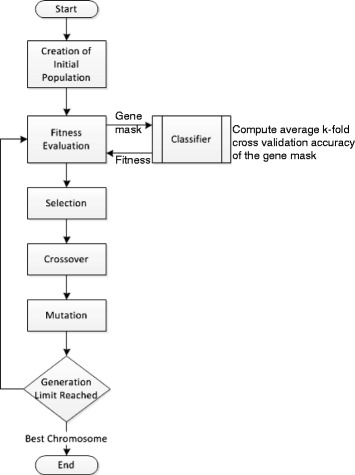
Flowchart depicting the relation of Genetic Algorithm and Classifier in gene masking where the best chromosome represents the best gene mask discovered

In essence, the basic purpose of GA in gene masking can be viewed as heuristically searching for the optimal gene mask that reduces the most features for a particular problem while maintaining high classification accuracy. The holistic approach taken when applying gene masking is shown in Fig. 2.

### Process details

**Fig. 3:**
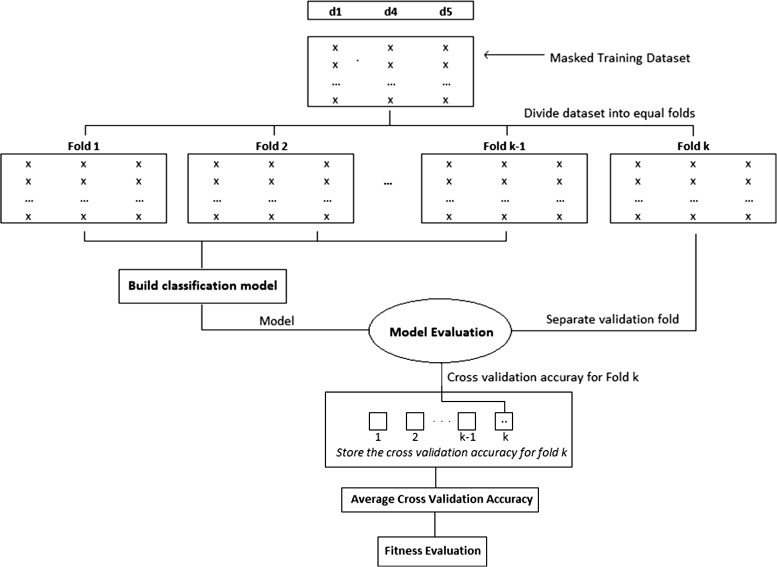
Illustration of fitness evaluation with gene masking. Cross validation is performed using a classifier and the average accuracy is used for fitness calculation

In order to determine the fitness of each mask, a classifier model is created using the masked dataset and its classification accuracy is evaluated using *k*-fold cross validation. The masked dataset is divided into *k* number of folds and a model is iteratively built using *k-1* folds and while the *k* ^th^ fold is isolated for model evaluation, yielding a set containing *k* classification accuracy values (one for each fold). Then, the fitness of a mask is computed based on its impact on classification accuracy while also considering the effective reduction in dimensionality. The details of fitness evaluation for gene masking is highlighted in Fig. 3, which describes intricacies between the classification algorithm and the masking process.

Upon fitness evaluation, GA goes through its orthodox set of operators, namely selection, crossover and mutation. Selection has been performed using roulette wheel selection, which is biased towards individuals with higher fitness. Crossover is accomplished by performing a random one-point binary crossover to swap the genes and mutation is performed by negating gene values at random locations. However, to preserve the highest performing chromosome between generations, elite selection is used to ensure that a mask with the highest fitness is passed to the next generation unmodified by GA operators.

The actual fitness value provided to GA is measured in terms of a weighted sum of the average classification accuracy from *k*-fold cross validation and the ratio of features removed from data, which is highlighted in Eq. 6. This sum is weighted using a constant *α*, called the *Accuracy to Elimination Ratio*, which is empirically chosen to direct the evolution of GA either towards attaining better classification accuracy or reducing the most number of features. The value of *α* is optimized within the interval (0, 1], where higher values of *α* give higher fitness values to masks with better accuracy while lower values of *α* give higher fitness values to masks with greater number of genes eliminated.

6 $$ Fitness = (Accuracy \times \alpha) + (1 - \alpha) \times \frac{Genes \; eliminated}{Total \; genes}   $$

This process of performing fitness evaluations and applying genetic operators continues until the number of generations specified during the initial parameter configuration is reached. The best chromosome discovered during the evolution of the population is selected. This chromosome represents the gene mask that yielded the highest fitness value during training. The best evolved gene mask is subsequently used for masking the test dataset during the testing phase.

## Experiment and discussion

Primarily, we had considered the SRBCT dataset for gene masking. The following sections provide details on the data, and the experiment and its results.

### Dataset

Gene masking was applied on the dataset containing gene-expression profiling using cDNA microarrays on small round, blue cell tumors (SRBCT) of childhood, named as such due to their similarity to routine histology. Each type of tumor can be classified into one of four classes either neuroblastoma (NB), rhabdomyosarcoma (RMS), non-Hodgkin lymphoma (NHL) or the Ewing family of tumors (EWS). The dataset comprises of 63 training samples and 25 test samples, each of which contains 2308 gene expressions from cDNA microarrays [1, 21]. Of the 25 test samples, 5 samples are not SRBCTs, which were discarded for the purpose of this study since corresponding non-SRBCT samples were not present in the training set. Classification by microarrays is a difficult task since the number of features (genes) are relatively large whereas the number of samples are relatively small and it is also important to identify genes that contribute most to classification [1, 21].

### Results

GA, and subsequently, gene masking, is stochastic by nature. During our experiment, multiple experiments with the same parameter combinations were executed while tuning GA parameters to get a consolidated view on the performance of gene masks with a particular combination of parameters.

As stated previously, gene masking is implemented by applying a mask to select a subset of features from data. GA is used to heuristically create masks (represented as a chromosome within GA) and evaluate their relative fitness. The parameters for GA were determined by empirical testing, whereby the population size was fixed to 105 and the chromosome length set to 2308 (the number of gene expressions in SRBCT dataset), and the best performing rates for crossover and mutation were determined to be 0.85 and 0.1 respectively. These initial parameter configurations were determined by experimentally evaluating the performance of GA with multiple experimental runs (around 10 runs for each combination of parameters) to produce a baseline from which the best parameter configurations were selected. The initial parameter configurations of GA are shown in Table 1. These simulations were conducted with *k*-fold cross validation for *k = 5*. The actual parameter tuning and selection procedure has been described in an algorithmic form in Table 2.

**Table 1:** Genetic algorithm parameters

Parameter	Value
GA type	Binary
Population size	105
Chromosome length	2308
No. of generations	50000
Selection function	Roulette wheel
Crossover rate	0.85
Mutation rate	0.10
Elite conservation	Yes, num_elite=1

**Table 2:** Parameter tuning and selection method used in this study

Parameter tuning and selection
Let *S* be the set of training samples
Let *CR* be the crossover rate and *MR* be the mutation rate
Let *k* be the number of cross validation folds, where *k* = 5 is fixed
Let *α* be the *Accuracy to Elimination Ratio*
Define the GA parameters apart from *CR* and *MR* as those highlighted in Table 1
Define *α* to belong to the set (0, 0.1, 0.2, …, 0.9, 1]
Define *CR* to belong to the set (0.5, 0.55, 0.6, …, 0.95, 1]
Define *MR* to belong to the set [0, 0.05, 0.1, …, 0.45, 0.5)
For each combination of { *α*, *CR*, *MR*}:
- Perform *k*-fold cross validation using the classifier and gene masking on the set of samples *S*
- Report the results obtained by the best performing *gene mask*
- Repeat for 10 iterations
Select the best performing combination of { *α*, *CR*, *MR*} for testing and reporting

During the initial phases of experiments, NCC was used with gene masking to evaluate the performance against the SRBCT dataset. This approach yielded good results with 100% classification accuracy, however, there was only about 28% reduction in genes (about 650 genes) from the original microarray data. This may be attributed to the fact that NCC is a very basic classifier. Additionally, it can be noted that with NCC, having a lower value for *α* (signifying a greater preference towards dimensionality reduction) yielded better results with *α*=0.3, giving 100% training and test accuracies.

The experiment was repeated by replacing NCC with NSCC whereby the results considerably improved. There was significant reduction in dimensionality while maintaining high classification accuracy. The best results with NSCC were shown with a solution comprising of 13 genes with 100% training and test accuracies. However, it must be stated that with NSCC, gene masking was performed on a “shrunken” dataset with about 70-120 genes depending on the value of *Δ*. The optimal range values for *Δ* that produced the best overall performance were in the interval of (6, 9] with steps of 0.5. Additionally, the optimal value that was observed for *α* was *α* *=0.9* signifying that a greater bias towards accuracy yielded better results with NSCC. The performance of gene masking with NSCC for varying values of *Δ* is shown in Table 3. The training accuracies for each of the reported samples in Table 3 was 100%. Additionally, a comparison of performance of gene masking with NCC and NSCC is highlighted in Table 4.

**Table 3:** Gene masking and NSCC performance on SRBCT test set with different values for *Δ* with *α* *= 0.9*

*Δ*	Genes left	Genes left	Test accuracy
	after shrinkage	after masking	
3	343	36	0.9
3.5	280	23	0.95
4	235	21	0.95
4.5	208	15	0.9
5	174	14	0.95
5.5	158	14	0.95
6	135	12	0.95
6.5	124	15	1
7	112	16	1
7.5	102	13	1
8	90	17	1
8.5	80	20	1
9	72	19	1
9.5	65	18	0.95
10	61	14	0.8
10.5	54	15	0.75
11	48	12	0.75
11.5	42	13	0.8
12	41	10	0.8

**Table 4:** Comparison of performance of NCC and NSCC with gene masking

	NCC	NSCC
Number of genes remaining	1637	13
Training accuracy	100%	100%
Test accuracy	100%	100%

NSCC removes features only on the basis of their magnitude of deviation of the classful means from the overall mean and, therefore, the interdependencies between features are not considered. Tibshirani et al. [21] used NSCC with the SRBCT dataset and identified 43 genes that lead to 100% classification accuracy. However, with gene masking, similar classification accuracy was achieved with only 13 genes. This can be attributed to the fact that gene masking eliminates genes based on their impact on classification, identifying major interdependencies between features and ensuring their survival during the evolution of gene masks. A comparison of results with similar techniques has been illustrated in Table 5.

**Table 5:** Comparison of performance of similar techniques

Method (Classifier)	Number of genes	Accuracy
PCA, MLP, Neural Network [1]	96	100%
Nearest Shrunken Centroid [21]	43	100%
Information gain + SVM [26]	150	95%
Towing rule + SVM [26]	150	95%
Sum minority + SVM [26]	150	95%
Max minority + SVM [26]	150	91%
Gini index + SVM [26]	150	95%
Sum of variances + SVM [26]	150	95%
t-statistics + SVM [26]	150	95%
One-dimensional SVM + SVM [26]	150	95%
Information gain + LDA with NCC [20]	4	70%
Chi-squared + NNC [20]	4	70%
Gain Ratio + NNC [20]	4	85%
Gene masking + ANN [22]	13	100%
Gene masking + NCC (this paper)	650	100%
Gene masking + NSCC (this paper)	13	100%

In NSCC, if the amount of shrinkage is kept relatively low (a lower value for *Δ*, which leaves more features in the dataset), gene masking is able to evaluate interdependencies between the remaining features. With the proposed technique, genes that were previously eliminated solely on the value of *Δ* are kept. Gene masking commences with around 100-120 genes, which are systematically evaluated and eliminated based on the gene masks produced by GA. Eventually, gene masking yields a solution with only 13 genes and as per the results shown in Table 6, it can be seen that only 6 of the genes discovered in the best solution of 13 genes belong to the 43 genes identified by Tibshirani et al. [21]. Also, it can be seen that majority of the genes identified by gene masking are also present in the 96 genes identified by Khan et al. [1]. Conversely, it can also be seen that this approach yields different results to those achieved by Kumar et al. [22], by noting the lack of any significant overlap between the identified genes.

**Table 6:** The 13 genes selected via gene masking with their relative occurrence in other solutions

Image	Name	Percentage	In [21]	In [1]	In [22]
ID		occurrence			
39093	methionine aminopeptidase;	42.86%	No	Yes	No
	eIF-2-associated p67				
365826	growth arrest-specific 1	100%	No	Yes	No
1416782	creatine kinase, brain	100%	No	Yes	No
461425	myosin MYL4	71.43%	Yes	Yes	No
810057	cold shock domain protein A	100%	Yes	No	No
866702	protein tyrosine phosphatase,	57.14%	Yes	Yes	Yes
	non-receptor type 13 (APO-1/CD95				
	(Fas)-associated phosphatase)				
854899	dual specificity phosphatase 6	28.57%	No	Yes	No
629896	microtubule-associated protein 1B	71.43%	No	Yes	Yes
214572	ESTs	100%	No	No	No
208718	annexin A1	100%	No	Yes	No
784224	fibroblast growth factor receptor	100%	Yes	Yes	No
204545	ESTs	57.14%	Yes	Yes	No
295985	ESTs	100%	Yes	Yes	No

Furthermore, due to the stochastic nature of gene masking, the gene masks that produce 100% accuracies do not tend to select the same combination of genes. Therefore, we have also identified and reported the relative occurrence of these genes (in Table 6) during various iterations where solutions that gave 100% accuracy with 15 genes or less were observed.

## Discussion

Gene masking can be very useful in feature selection and it can isolate features that lead to high classification accuracy. As per the results on the SRBCT dataset, it can be seen that gene masking can be used to identify features which have significant contribution towards classification.

However, in order to further investigate the proposed technique, gene masking in conjunction with NSCC was used to classify even larger datasets (in terms of number of genes in gene expression data). These datasets were mixed-lineage leukemia (MLL) [23] and lung cancer (LC) [24] datasets. The MLL dataset comprises of 12,582 gene expressions for each sample. It consists of 57 training samples and 15 test samples and each of these samples can be categorized into one of three cancer types, either ALL, MLL or AML [23]. On the other hand, LC dataset contains tissue samples of two cancer types, MPM or ADCA, consisting of 32 training samples and 149 test samples with each sample comprising of 12,533 genes expressions [24].

With these sets of data, gene masking was able to produce 100% training and test accuracy when the datasets were shrunk to about 400 genes using NSCC and gene masking was able to further reduce and isolate about 90 genes each. These results are highlighted in Table 7. All parameters used in these sets of experiments remained similar to those stated earlier.

**Table 7:** A summary of performance of gene masking with NSCC on MLL Leukemia and Lung Cancer datasets

Dataset	Genes remaining	Test accuracy
MLL Leukemia	94	100%
Lung Cancer	90	100%

It should be noted that gene masking has been derived completely off a basic binary GA. As with most evolutionary global optimization algorithms, the risk of getting stuck in local optima is greater when the search space is extremely large. While searching for global optimal locations in a large search domain, a subsequent degradation in performance can be noted. Gene masking currently suffers from a similar limitation, which is highlighted by the results summarized in Table 7 for MLL and LC datasets.

Even with NSCC as the classifier that allows for an “in-built” feature selection procedure, the performance of gene masking was not as good as those with the SRBCT dataset, if dimensionality reduction is considered as a basis of performance. If the amount of shrinkage by NSCC is increased, there is a lot of loss of information solely on the basis of the magnitude of variation from the overall mean without considering feature interdependencies. Therefore, with NSCC, MLL and LC datasets could only be shrunk to about 400 genes each prior to initializing gene masking. From there onwards, gene masking was able to further reduce the number of genes required to maintain 100% accuracy to about 90 genes for both datasets.

## Conclusion

Gene masking can be very useful in feature selection as it can isolate features that lead to high classification accuracy. It does so by considering the impact of features on classification and heuristically removes non-contributing features. In this paper, we have demonstrated its viability by achieving 100% accuracy while significantly reducing the number of genes required on SRBCT, MLL and LC datasets containing microarray gene expressions for cancers.
